# Homeoffice spart ein Zehntel Treibstoff ein

**DOI:** 10.1007/s10273-022-3228-y

**Published:** 2022-07-23

**Authors:** Oskar Jost, Holger Seibert

**Affiliations:** IAB Berlin-Brandenburg, Friedrichstraße 34, 10969 Berlin, Deutschland

**Keywords:** J61, R23, R40, R41

## Abstract

Aufgrund der rapide gestiegenen Treibstoffpreise wird die Debatte zu möglichen Einsparpotenzialen bei Treibstoff wichtiger. So könnten die in der Coronapandemie entwickelten Homeofficepotenziale dazu genutzt werden, um Pendelaufkommen zu reduzieren und so den Treibstoffverbrauch zu verringern. Um Einsparpotenziale zu bestimmen, wird eine für den deutschen Raum vorliegende Untersuchung genutzt, die Homeofficepotenziale für einzelne Berufsfelder ausweist. Diese Potenziale werden mit der Beschäftigungsstatistik der Bundesagentur für Arbeit kombiniert. Diese Statistik liefert erste berufsspezifische Informationen zu Pendeldistanzen der Beschäftigten, die über Gemeindegrenzen hinweg pendeln und so besonders von hohen Treibstoffpreisen betroffen sind.

## Abstract (en)

In light of the sharp rise in fuel prices, this article takes up an important discussion on potential fuel savings. We argue that the work from home capacity, which developed during the COVID-19 pandemic, could be used to reduce commuting and thus fuel consumption. In order to determine the amount of fuel savings, we use a study available for Germany that provides information on work from home capacities for different occupations. Further, we merge this information with the employment statistics of the Federal Employment Agency providing information on occupations and commuting. This approach enables us to identify employees working and living in different municipalities, which are particularly affected by sharp rises in fuel prices.

Laut Kraftfahrtbundesamt (KBA) haben die Deutschen im Jahr 2020 zusammen gut 626 Mrd. Kilometer in Pkw zurückgelegt. Im Jahr 2019 waren es mit 632 Mrd. Kilometern nur unwesentlich mehr (KBA, 2021). Bei einem Durchschnittsverbrauch von 7,4 Litern je 100 Kilometer (BMVI, 2021) entspricht dies gut 46 Mrd. Litern Treibstoff. Mit dem Ukrainekrieg haben sich die Treibstoffpreise noch einmal deutlich verteuert, nachdem sie ohnehin bereits auf einem höheren Niveau lagen als in den Jahren zuvor. Ein großer Teil der Jahreskilometerleistung von Pkw geht auf den Pendelverkehr zur Arbeit zurück. Zudem zeigt das Statistische Bundesamt (2022), dass knapp 70 % aller Pendler:innen das Auto für den Weg zur Arbeit nutzen. Gerade hier bestünde ein sichtbares Einsparpotenzial für Treibstoffe, wenn Beschäftigte, die die Möglichkeit haben, von zu Hause aus zu arbeiten und dies wie schon in den Lockdownphasen der Coronapandemie erneut verstärkt tun würden. Sie würden so an den Homeofficetagen den Arbeitsweg nicht mit dem Auto fahren müssen.

**Oskar Jost** und **Dr. Holger Seibert** sind wissenschaftliche Mitarbeiter am Institut für Arbeitsmarkt- und Berufsforschung (IAB), Forschungsbereich Regionales Forschungsnetz, in Berlin.

Unsere Modellrechnung soll eine Diskussion anregen, mit der Frage, welches Einsparpotenzial verstärktes Homeoffice zum Treibstoffverbrauch beitragen könnte. Es werden dabei mehrere Varianten (Quoten) der Nutzung von Homeoffice durch Beschäftigte unterstellt und dafür die Pendlerdaten der Beschäftigungsstatistik der Bundesagentur für Arbeit auf Gemeindeebene für den Berichtsmonat Juni 2021 ausgewertet. Mithilfe dieser Daten ist es möglich, Distanzen zwischen den Arbeits- und Wohnorten der Beschäftigten zu ermitteln, die zwischen zwei verschiedenen Gemeinden zur Arbeit pendeln. Zudem wird auf eine vorliegende Studie (Alipour et al., 2021) zurückgegriffen, die die Möglichkeiten zur Homeofficenutzung berufsspezifisch differenziert. Beispielsweise können Büroberufe in einem größeren Umfang im Homeoffice erledigt werden als Berufe im Handwerk oder in der industriellen Produktion.

## Daten und Einschränkungen

Für unsere Modellrechnung nutzen wir die Daten der Beschäftigungsstatistik. Neben den Arbeits- und Wohnortgemeinden enthält die Beschäftigungsstatistik auch die Berufe der Beschäftigten, sodass das Einsparpotenzial von Fahrten zur Arbeit über berufsspezifische Homeofficequoten näher bestimmt werden kann. In den Beschäftigungsdaten liegen lediglich die Gemeinden als Ortsinformation vor, aber keine konkreten Adressen. Daher werden die Distanzen zwischen Wohn- und Arbeitsort annäherungsweise über die Distanzen zwischen den jeweiligen geografischen Gemeindemittelpunkten (geometrisches Zentrum der Gemeindefläche) und ein anschließendes Routingverfahren ermittelt[1], indem die kürzeste Distanz über das öffentliche Straßennetz bestimmt wird. Dieses Verfahren birgt ei-

1 Zur Berechnung der Distanzen zwischen den Gemeindemittelpunkten wurde der Algorithmus von Huber und Rust (2016) verwendet.

ne gewisse Unschärfe, da die Pendeldistanzen aufgrund fehlender Berücksichtigung des Pendelns innerhalb von Gemeinden überschätzt werden. Gleichwohl sollte sich diese potenzielle Überschätzung der Distanzen nicht auf eine grundsätzliche Diskussion zu diesem wichtigen Thema auswirken. Einerseits zeigen Forschungspapiere, dass eine Analyse des Pendelns über Gemeinden hinweg eine gute Approximation der Mobilitätsdynamik bietet (Meekes und Hassink 2019; Duan et al., 2022). Andererseits haben Beschäftigte bei der innerstädtischen Mobilität eher die Möglichkeit, auf Alternativen zum Auto umzusteigen. Ihr Arbeitsweg dürfte zumeist deutlich kürzer sein und in größeren Städten steht zudem ein besser ausgebauter öffentlicher Personennahverkehr zur Verfügung. Eine Ausweitung der Homeofficetätigkeit würde für den innerstädtischen Verkehr also geringere Einsparpotenziale mit sich bringen als für die Mobilität zwischen Gemeinden.

## Methodik

Entsprechend der zuvor genannten Einschränkungen, konzentrieren wir uns ausschließlich auf Beschäftigte, die über Gemeindegrenzen hinweg pendeln, die wir nachfolgend als mobile Beschäftigte bezeichnen. Demnach waren im Juni 2021 von den knapp 34 Mio. sozialversicherungspflichtig Beschäftigten in Deutschland über 20 Mio. in einer Gemeinde außerhalb ihres Wohnorts beschäftigt.

Abbildung 1

**Berufshauptgruppen mit höchstem und niedrigstem Homeofficepotenzial, Deutschland, Juni 2021**

in %

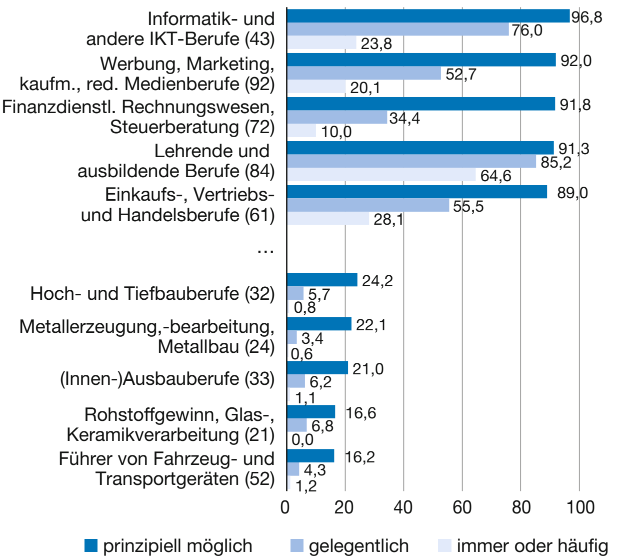

Sortierung der Berufshauptgruppen nach der Kategorie „prinzipiell möglich“.

Quelle: Statistik der Bundesagentur für Arbeit; Alipour et al. (2021); eigene Darstellung.

Alipour et al. (2021) haben basierend auf der Arbeitskräfteerhebung der BIBB-/BAuA-Befragung ermittelt, dass 2018 ca. 9 % der Erwerbstätigen immer oder häufig von zu Hause aus arbeiten. Gelegentlich von zu Hause aus zu arbeiten, gaben 26 % der Befragten an. Für ca. 56 % ist eine Arbeit von zu Hause zumindest möglich, wenn der Arbeitgebende diese ermöglichen würde. Der letzte Wert stellt damit eine theoretische Obergrenze der realisierbaren Homeofficekapazitäten dar. Ferner haben Alipour et al. (2021) die Homeofficepotenziale für die verschiedenen Berufshauptgruppen differenziert ermittelt.[2] Diese berufsspezifischen Homeofficepotenziale wurden bereits für verschiedene regionale Untersuchungen zu den sogenannten systemrelevanten Berufen und ihrem Potenzial für Homeoffice genutzt (Otto et al., 2021a; Otto et al., 2021b; Fuchs et al., 2021a/b).

Die ermittelten Homeofficepotenziale fallen je nach Beruf unterschiedlich aus. Abbildung 1 zeigt exemplarisch die Homeofficeintensität in den jeweils fünf Berufshauptgruppen mit den höchsten bzw. niedrigsten Möglichkeiten, von zu Hause aus zu arbeiten. Die Liste wird angeführt von den Informatikberufen, in denen nahezu komplett von zu Hause aus gearbeitet werden kann. Demnach arbeiteten zum Befragungszeitpunkt 2018 ein knappes Viertel dieser Erwerbstätigen bereits häufig im Homeoffice, gut drei Viertel gelegentlich. Besonders niedrig fällt das Homeofficepotenzial hingegen bei der Führung von Fahrzeug- und Transportgeräten aus. Hier dürfte sich das Arbeiten von zu Hause im Wesentlichen auf die Arbeitsvorbereitung beschränken. Einen Sonderfall mit vermeintlich besonders hohen Homeofficepotenzialen stellen die Lehrenden dar. Zwar haben sie in den Lockdownphasen der Coronakrise unter Beweis stellen müssen, dass Unterrichten auch von zu Hause aus über Konferenzsysteme und entsprechende Lernplattformen möglich ist. In der Regel ist diese Arbeit aber zumindest an den allgemeinbildenden und beruflichen Schulen durch die Arbeitsorte Schule (Unterricht) und zu Hause (Unterrichtsvorbereitung) charakterisiert. Dementsprechend geben diese Befragten zwar an, häufig von zu Hause aus zu arbeiten, gleichzeitig müssen sie aber genauso oft persönlich in der Schule anwesend sein.

Werden die Informationen zu den berufsspezifischen Pendeldistanzen auf Basis der Beschäftigungsstatistik herangezogen, ergeben sich auch hier erkennbare Unterschiede

2 Zu beachten ist bei den Analysen von Alipour et al. (2021) jedoch, dass keine Informationen darüber vorliegen, ob die Tätigkeiten tatsächlich komplett von zu Hause aus erledigt werden können. So bleibt unberücksichtigt, ob zur häufigen Homeoffice-Möglichkeit auch eine regelmäßige Anwesenheit am Arbeitsplatz erforderlich ist, wie etwa im Fall der Lehrenden.

Abbildung 2

**Berufshauptgruppen mit höchsten und niedrigsten Pendeldistanzen, Deutschland, Juni 2021**

in Kilometer

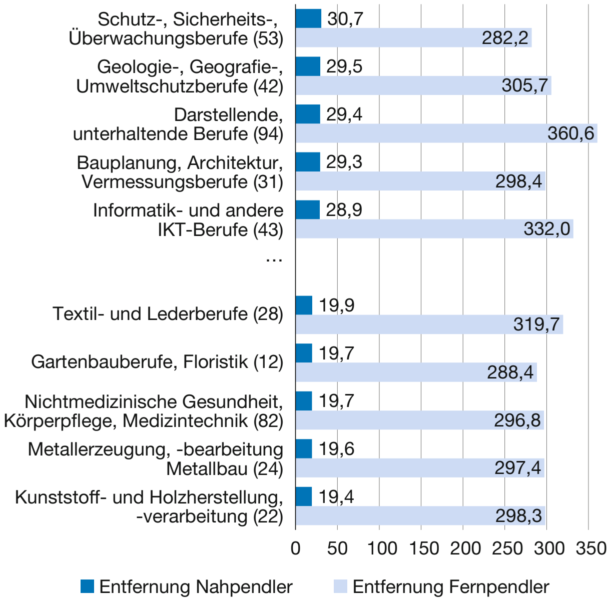

Sortierung der Berufshauptgruppen nach den durchschnittlichen Entfernungen der Nahpendler.

Quelle: Beschäftigungsstatistik der Bundesagentur für Arbeit, eigene Darstellung.

(vgl. Abbildung 2). Zusätzlich unterteilen wir in Abbildung 2 die Pendelnden in Nahpendelnde (bis 100 km für die einfache Strecke) und Fernpendelnde (über 100 km) und ihre jeweiligen durchschnittlichen Distanzen. Wir folgen dabei Dauth und Haller (2018) sowie Duan et al. (2022) und unterstellen, dass einfache Strecken bis 100 km täglich und Strecken über 100 km nur einmal in der Woche gefahren werden (Wochenpendler:innen). Demnach beträgt die durchschnittliche einfache Pendeldistanz der nahpendelnden Beschäftigten in den Schutz-, Sicherheits- und Überwachungsberufen 30,7 km. Hingegen weisen Fernpendelnde in diesen Berufen eine durchschnittliche Pendeldistanz von rund 282 km auf. Besonders niedrig ist mit 19,4 km der Weg zur Arbeit bei nahpendelnden Beschäftigten in den Berufen der Kunststoff- und Holzherstellung und -verarbeitung (Fernpendelnde rund 298 km). Zudem zeigt sich, dass Berufe, in denen Fernpendelnde besonders lange Entfernungen zurücklegen müssen, wie in den darstellenden, unterhaltenden oder IT-Berufen, sich überwiegend in städtischen Räumen finden, für die aus dem Umland weitere Wege anfallen.

Bringt man nun die personengewichteten berufsspezifischen Pendeldistanzen mit den berufsspezifischen Homeofficepotenzialen zusammen, kann man Treibstoffbedarfe für verschiedene Szenarien berechnen. Um eine

Tabelle 1

**Nah- und Fernpendler:innen sowie durchschnittliche Entfernung innerhalb Deutschlands, Juni 2021**

| | Nahpendler:innen (<=100 km für die einfache Fahrt) | Fernpendler:innen (>100 km für die einfache Fahrt) |
|---|---|---|
| Zahl in 1.000 | 18.003 | 2.107 |
| durchschnittliche Entfernung (hin und zurück in km) | 23,5 | 313,6 |

Quelle: Beschäftigungsstatistik der Bundesagentur für Arbeit, eigene Berechnungen.

Vorstellung von der Größenordnung des Einsparpotenzials durch das Arbeiten von zu Hause zu erlangen, sind Annahmen über die Zahl der Tage pro Jahr erforderlich, an denen Pendelnde zur Arbeit fahren bzw. dies unterlassen und komplett von zu Hause aus arbeiten. Da hierzu keine Informationen vorliegen, werden im Folgenden einige Annahmen und Szenarien diskutiert. Zunächst unterscheiden wir, ob die Pendelnden entsprechend ihrer jeweiligen Distanz zwischen Wohn- und Arbeitsort zu Nahpendelnden bzw. Fernpendelnden gezählt werden. Wie Tabelle 1 zeigt, gibt es im Juni 2021 in Deutschland ca. 18 Mio. Nahpendelnde, die im Schnitt 23,5 Kilometer für die einfache Strecke zurücklegen. Die gut 2,1 Mio. Fernpendler:innen legen im Schnitt 313,6 Kilometer für eine Strecke zurück. Für die weitere Berechnung gehen wir von der Annahme aus, dass Nahpendelnde fünfmal pro Woche und damit unter Berücksichtigung der Urlaubszeiten an 230 Tagen im Jahr zur Arbeit fahren, Fernpendelnde hingegen nur einmal pro Woche und damit an 46 Tagen im Jahr. Für Fernpendelnde ist aufgrund der hohen Entfernung davon auszugehen, dass diese eine Unterkunft am Arbeitsort nutzen, um nicht täglich fahren zu müssen.

Legt man diese Annahmen zugrunde, ergibt sich für die gut 20 Mio. Pendelnden, die über Gemeindegrenzen hinweg mobil sind, eine jährliche Gesamtkilometerzahl von ca. 256 Mrd. Kilometern (Tabelle 2). Daraus lässt sich wiederum der Jahrestreibstoffverbrauch für alle Pendelnden, die den Pkw nutzen, errechnen sowie für bestimmte Homeofficeintensitäten. Der Jahresverbrauch wird dabei unter der Annahme errechnet, dass 70 % der Pendelnden mit dem Auto zur Arbeit fahren (Statistisches Bundesamt, 2022) und im Schnitt 7,4 Liter Treibstoff auf 100 Kilometer verbrauchen (BMVI, 2021). Damit beziehen sich die weiteren Überlegungen auf gut 14 Mio. Pendelnde, die mit dem Pkw fahren. Die Verbrauchswerte für die verschiedenen Homeofficeintensitäten bilden dabei (theoretische) Einsparpotenziale unter den verschiedenen Annahmen ab. Unter den beschriebenen Annahmen ergibt sich ein Jahrestreibstoffverbrauch für Fahrten zur Arbeit und zurück mit dem Auto von 13,28 Mrd. Litern Treibstoff.

Wenn Pendelnde mit Berufen, in denen immer oder häufig von zu Hause aus gearbeitet werden kann, ganzjährig – also an 230 Arbeitstagen – komplett von zu Hause aus arbeiten würden und damit keine Fahrten zur Arbeit zurücklegen müssten, würde sich der Gesamtverbrauch um 1,31 Mrd. Liter verringern. Würden alle Pendler:innen, deren Berufe ein Arbeiten von zu Hause prinzipiell ermöglichen, an allen 230 Arbeitstagen im Jahr von zu Hause aus arbeiten, würde sich der Treibstoffverbrauch rechnerisch um 7,64 Mrd. Liter reduzieren und sich damit mehr als halbieren. Ein solches Szenario ist allerdings sehr unrealistisch. Wenn man davon ausgeht, dass Pendelnde in Berufen, in denen immer oder häufig im Homeoffice gearbeitet wird, an durchschnittlich 100 Tagen komplett von zu Hause arbeiten, wäre der Jahrestreibstoffverbrauch damit bereits um mehr als ein halbe Milliarde Liter reduziert. Würden diese Pendelnden ihre Homeofficeaktivität auf 200 Arbeitstage verdoppeln, könnten 1,14 Mrd. Liter eingespart werden.

Würden zudem Pendelnde mit Berufen, in denen gelegentlich von zu Hause aus gearbeitet wird, an 50 Tagen komplett im Homeoffice arbeiten, hätte dies ein Einsparpotenzial von 0,78 Mrd. Litern Treibstoff, bei 100 Tagen im Homeoffice sogar von 1,57 Mrd. Zusammen wäre das bereits eine Ersparnis von 1,35 Mrd. bzw. 2,71 Mrd. Litern Treibstoff. Das entspricht 10,2 % bzw. 20,4 % der Gesamtmenge von 13,28 Mrd. Litern, die für die Pkw-Fahrten der Pendelnden über Gemeindegrenzen hinweg rechnerisch erforderlich wären. Das tatsächliche Einsparpotenzial dürfte jedoch niedriger ausfallen, da ein bestimmter Teil der Pendelnden bereits heute einen gewissen Teil seiner Arbeitszeit im Homeoffice erbringt oder Mitfahrgelegenheiten nutzt und daher der rechnerische Gesamttreibstoffverbrauch von gut 13 Mrd. Litern nicht ausgeschöpft werden dürfte. Auf der anderen Seite kommen aber gegebenenfalls bei Fernpendler:innen, die in unseren Berechnungen nur mit einer einmaligen Hin- und Rückfahrt pro Arbeitswoche berücksichtigt sind, noch Fahrten zwischen der Zweitunterkunft am Arbeitsort und der Arbeitsstätte hinzu, die wegfallen würden.

Eine stärkere Ausweitung der Homeofficetätigkeit als in den oben skizzierten Szenarien könnte zumindest rechnerisch zu einem noch deutlich größeren Ersparnis von Treibstoffen für den Weg zur Arbeit führen. Gleichzeitig könnte es sogar gezielt als politisches Instrument im Falle von plötzlich eintretenden akuten Treibstoffengpässen, etwa infolge eines Ölembargos, eingesetzt werden. Außerdem würden weniger Fahrten zur Arbeit auch eine deutlich finanzielle Entlastung der Beschäftigten mit sich bringen. Bei aktuellen Treibstoffpreisen von ca. 2 Euro pro Liter würden die beiden oben genannten Einsparpotenziale von 1,35 Mrd. bzw. 2,71 Mrd. Litern Treibstoff die Pkw-Pendelnden rechnerisch um 2,70 Mrd. bzw. 5,42 Mrd. Euro entlasten.

Tabelle 2
**Gesamtpendeldistanz und erforderlicher Treibstoffbedarf für Pkw-Fahrten zur Arbeit**

| | Alle Pendler über Gemeindegrenzen | Pendler nach Homeoffice-Intensität (nach Alipouret al. 2021) | | |
|---|---|---|---|---|
| | | immer oder häufig | gelegentlich | prinzipiell möglich |
| | (1) | (2) | (3) | (4) |
| Beschäftigung | | | | |
| Zahl in 1.000 | 20.110 | 1.902 | 5.288 | 11.399 |
| Prozent | 100 | 9,5 | 26,3 | 56,7 |
| Gesamtkilometer pro Jahr: | | | | |
| Nahpendelnde an 230 Arbeitstagen im Jahr (hin und zurück, im Durchschnitt 46,9 km) Fernpendelnde an 46 Tagen (1x pro Arbeitswoche) im Jahr (hin und zurück im Durchschnitt 627,2 km) | | | | |
| in Mrd. | 256 | 25 | 70 | 148 |
| Jahrestreibstoffverbrauch bei 70 % Pkw-Anteil und einem Durchschnittsverbrauch von 7,4 l/100 km | | | | |
| 230 Arbeitstage in Mrd. L | 13,28 | 1,31 | 3,61 | 7,64 |
| 200 Arbeitstage in Mrd. L | | 1,14 | 3,14 | 6,65 |
| 150 Arbeitstage in Mrd. L | | 0,86 | 2,35 | 4,98 |
| 100 Arbeitstage in Mrd. L | | 0,57 | 1,57 | 3,32 |
| 50 Arbeitstage in Mrd. L | | 0,29 | 0,78 | 1,66 |

Quelle: Alipour et al. (2021); Beschäftigungsstatistik der Bundesagentur für Arbeit; eigene Darstellung.

## Fazit

Dieser Beitrag hat zum Ziel, eine Diskussion um das Pendeln anzustoßen. Obwohl unsere Berechnung von vielen Einflussfaktoren, Abhängigkeiten oder Zusammenhängen abstrahiert, können wir erste Anhaltspunkte zur Größenordnung der potenziellen Einsparmöglichkeiten liefern. Zu berücksichtigen ist, dass wir hier lediglich die Gruppe der mobilen Beschäftigten, die zwischen verschiedenen Gemeinden pendeln, betrachtet haben. Damit unterschätzen wir das Einsparpotenzial, da wir den Pendelverkehr innerhalb der Gemeinden unberücksichtigt lassen.

Weite Wege zur Arbeit gehen zwar häufig mit besseren Lohn- und Arbeitsangeboten einher, bringen aber auch hohe Kosten mit sich. Dabei sind es nicht nur die monetären Kosten, die für die Beförderung anfallen, sondern auch entgangene Freizeit und Familienzeit. Hier könnten Arbeitnehmende also in mehrfacher Hinsicht entlastet werden. Zusätzlich würde auch die Verkehrsinfrastruktur durch einen geringeren Berufsverkehr entlastet, was nicht nur positive Auswirkungen auf das Klima, sondern auch auf die Lebensbedingungen in den Ballungsräumen hätte. Schließlich wird durch jede vermiedene Autofahrt nicht nur der Schadstoffausstoß, sondern auch die Lärmemission reduziert und am nicht angefahrenen Zielort ein Parkplatz weniger verbraucht.

Weitere Beiträge und Forschung zu diesem Thema könnten zum einen eine detailliertere und präzisere Berechnung der Einsparpotenziale vornehmen und zum anderen die Thematik um weitere wichtige Punkte ergänzen. Hier ist insbesondere ein Fokus auf den ländlichen Raum sinnvoll, der durch steigende Homeofficepotenziale attraktiver wird und wo zudem Mieten und Wohneigentum günstiger sind als in den Ballungsräumen. Ähnliches gilt auch für potenzielle Folgeentwicklungen wie von Marz (2022) diskutiert, wonach Beschäftigte mit der Möglichkeit von zu Hause aus zu arbeiten ins Umland migrieren und so langfristig ihre Emissionen erhöhen, weil beispielsweise längere Fahrten im Umland zu erwarten sind.

**Title:** ***Home Office Saves a Tenth of Fuel***
